# Suboptimal LDL-C Goal Attainment After Ischemic Stroke and TIA: Prevalence, Determinants, and Clinical Implications

**DOI:** 10.3390/clinpract15110193

**Published:** 2025-10-23

**Authors:** Pawonrath Rabob, Arom Jedsadayanmata

**Affiliations:** 1 Graduate Program in Drug Utilization and Health Outcomes Research, Faculty of Pharmacy, Thammasat University, Khong Luang, Pathum Thani 12120, Thailand; pawonrath.rab@dome.tu.ac.th; 2 Faculty of Pharmacy, Thammasat University, Khong Luang, Pathum Thani 12120, Thailand

**Keywords:** atherosclerotic cardiovascular disease, goal attainment, ischemic stroke, low-density-lipoproteins, statin

## Abstract

**Background:** Achieving low-density lipoprotein cholesterol (LDL-C) goals is essential for secondary prevention after atherosclerotic ischemic stroke or transient ischemic attack (TIA). This study assessed the prevalence of LDL-C goal attainment and identified associated determinants post-ischemic stroke/TIA. **Methods:** A cohort of Thai patients discharged on statin therapy after admission with acute ischemic stroke or TIA was evaluated for LDL-C goal attainment within 12 months post-discharge. Logistic regression determines factors associated with LDL-C goal attainment, and the generalized linear model confirmed the association between covariates and LDL-C reduction. **Results:** Among 487 patients (85.8% with ischemic stroke), the prevalence of LDL-C goal attainment differed across LDL-C target levels: 9.9% for <55 mg/dL, 29.0% for <70 mg/dL, 70.4% for <100 mg/dL, and 17.5% for ≥50% reduction from baseline. Logistic regression identified high-intensity statins as a significant predictor of goal attainment at <70 mg/dL (OR 1.91, 95% CI 1.09–3.34), <100 mg/dL (OR 1.64, 95% CI 1.01–2.67), and ≥50% reduction (OR 2.32, 95% CI 1.14–4.73), but not <55 mg/dL (OR 1.65, 95% CI 0.72–3.79). In the generalized linear model, high-intensity statin and baseline LDL-C were significant determinants of LDL-C reduction. **Conclusions:** LDL-C target attainment after ischemic stroke/TIA is modest overall, and remains low for the more stringent targets (<55 mg/dL). High-intensity statins improve goal attainment and produce greater LDL-C reductions, supporting wider use of more aggressive lipid-lowering strategies in this high-risk population.

## 1. Introduction

Stroke is the second leading cause of disability and mortality worldwide, with ischemic stroke accounting for more than 80% of all stroke cases [1]. Ischemic stroke is a major atherosclerotic cardiovascular disease (ASCVD) for which elevated low-density lipoprotein cholesterol (LDL-C) is a target for secondary prevention [2,3]. Studies have shown that aggressive reduction in LDL-C with high-intensity statins provides benefits for secondary prevention after ischemic stroke or transient ischemic attack (TIA) [4]. Furthermore, minor ischemic stroke/TIA patients with an LDL-C level greater than 100 mg/dL who did not receive lipid-lowering therapy had a higher risk of ischemic stroke within the first three months [5].

Achieving LDL-C goals provides significant clinical benefits for patients with ischemic stroke or TIA. Previous studies have demonstrated that maintaining LDL-C levels below 70 mg/dL is more effective in reducing the risk of major cardiovascular events than an LDL-C goal range of 90 to 110 mg/dL [6]. Additionally, in patients with a goal LDL-C level below 70 mg/dL, a reduction in LDL-C by ≥50% from baseline is associated with a lower risk of major cardiovascular events compared to those with a target LDL-C range of 110 ± 10 mg/dL [7]. Furthermore, a meta-analysis has shown that reducing LDL-C by more than 50% from baseline significantly decreases the risk of recurrent ischemic stroke [8].

The attainment of the LDL-C goal in patients with ischemic stroke/TIA may remain suboptimal. A cross-sectional study in China reported that only 27% of these patients achieved an LDL-C level below 70 mg/dL [9]. In another study from Israel, 42% and 23% of patients with a history of ischemic stroke/TIA reached the LDL-C goal of <70 and <55 mg/dL, respectively [10]. These findings highlight the low rates of LDL-C goal attainment in patients with ischemic stroke/TIA.

High-intensity statins have been shown to influence LDL-C goal attainment in patients with ASCVD [11]. Among patients with acute coronary syndromes, high-intensity statin was associated with achieving LDL-C goals [12]. However, real-world evidence regarding the effectiveness of high-intensity statins in achieving LDL-C goals in patients with ischemic stroke/TIA remains limited. Therefore, the present study aimed to determine the prevalence of LDL-C target attainment and identify factors associated with achieving the LDL-C goal among patients discharged with acute ischemic stroke/TIA. The findings of this study may inform policymakers to improve the quality of care for this patient population.

## 2. Methods

### 2.1. Study Setting and Participants

This retrospective cohort study was conducted at a public general hospital in the central region of Thailand. The hospital serves as a referral center for community hospitals and primary care clinics, providing medical, surgical, and emergency care. It primarily serves a mixed patient population, including residents from rural communities and those from semi-urban areas with access to larger medical centers. The hospital operates under Thailand’s universal health coverage system, ensuring broad accessibility to healthcare, particularly for underserved populations in the region.

Patients hospitalized with acute ischemic stroke or TIA were identified using the International Classification of Diseases, Tenth Revision (ICD-10) codes: I63.0, I63.2, I63.5, I63.9, and G45.9. Eligible patients were those aged 18 years or older admitted to the study hospital between January 2019 and December 2022. Exclusion criteria included a diagnosis of atrial fibrillation, valvular heart disease, or a history of valve replacement. Additionally, patients were excluded if they were discharged with oral anticoagulants, were not prescribed statins at discharge, lacked an LDL-C measurement on index admission, or had an LDL-C measurement within the three months preceding admission. Eligible discharged ischemic stroke/TIA patients were further excluded from the analytic cohort if they had no LDL-C measurement within 12 months post-discharge.

### 2.2. Variables and Data Collection

Patient data were extracted from the hospital’s electronic medical records using a case record form approved by the Research Ethics Committee. The collected data included patient demographics, principal diagnosis, comorbidities, medications prescribed at discharge, and LDL-C levels at index admission and follow-up within 12 months after discharge. No personal identifiers were collected to protect patient confidentiality.

Statin therapy prescribed at discharge was categorized as high-, moderate-, or low-intensity in accordance with the 2019 European Society of Cardiology/European Atherosclerosis Society (ESC/EAS) guideline, based on the expected percentage reduction in LDL-C. High-intensity statins are defined as those expected to lower LDL-C by approximately 50% or more, moderate-intensity statins by 30–50%, and low-intensity statins by less than 30%. In this study, high-intensity therapy consisted of atorvastatin 40 mg/day, which was the highest dose prescribed at the study institution. Moderate-intensity therapy included atorvastatin 20 mg/day, simvastatin 40 mg/day, and simvastatin 20 mg/day, while low-intensity therapy comprised simvastatin 10 mg/day. Rosuvastatin was not available in the hospital formulary, and atorvastatin 80 mg was not prescribed during the study period.

LDL-C goal attainment was defined according to professional guidelines: LDL-C levels of <55 mg/dL, <70 mg/dL, or <100 mg/dL, or a reduction of at least 50% from baseline LDL-C at index admission [2,3]. The first LDL-C measurement taken at least one month after discharge and within the 12-month follow-up period was used to assess LDL-C goal achievement.

The time from discharge to the first LDL-C measurement was summarized using the mean, median, and predefined time windows (1–3, 4–6, and 7–12 months). LDL-C goal attainment was evaluated within each corresponding 3-, 6-, and 12-month post-discharge window. For each time frame, the denominator included all patients who had an LDL-C test up to that time point, and the numerator included those who achieved the specified LDL-C goal. The prevalence of goal attainment was calculated as follows:

$$\%\mathrm{LDL}-C\;\mathrm{goal}\;\mathrm{attainment}=\frac{\mathrm{Number}\;\mathrm{of}\;\mathrm{patients}\;\mathrm{achieving}\;\mathrm{LDL-C}\;\mathrm{goal}\;\mathrm{up}\;\mathrm{to}\;\mathrm{the}\;\mathrm{specified}\;\mathrm{time}}{\mathrm{Total}\;\mathrm{number}\;\mathrm{of}\;\mathrm{patients}\;\mathrm{with}\;\mathrm{LDL-C}\;\mathrm{tested}\;\mathrm{up}\;\mathrm{to}\;\mathrm{the}\;\mathrm{specified}\;\mathrm{time}}\times \;100$$

Prevalence estimates were reported as proportions with 95% confidence intervals (CIs).

### 2.3. Statistical Analyses

Continuous variables were summarized as means (standard deviations) or medians (interquartile ranges (IQR)), and categorical variables as frequencies (percentages).

Determinants of LDL-C goal attainment were identified using multivariable logistic regression, with results expressed as adjusted odds ratios (ORs) and 95%CIs. A literature review identified potential predictor variables for attaining the LDL-C goal, including gender, age, baseline LDL-C, smoking status, body mass index (BMI), coronary artery disease (CAD), diabetes mellitus (DM), chronic kidney disease (CKD), statin intensity, and use of other cholesterol-lowering agents. The independent variables in the logistic regression included: age, gender, smoking status, DM, CKD, CAD, and baseline LDL-C. Collinearity among covariates was evaluated using the variance inflation factor (VIF), with a VIF greater than five indicating multicollinearity, which led to the exclusion of the affected variable.

We further confirmed the association between determinants and the percentage change in LDL-C within 12 months using a generalized linear model (GLM) with a Gaussian distribution and an identity link function. The model included gender, age, DM, CKD, CAD, smoking status, and baseline LDL-C as covariates. To account for potential heteroscedasticity, we applied heteroscedasticity-robust standard errors using the Huber-White sandwich estimator to ensure valid inference. To formally assess heteroscedasticity, we regressed Pearson residuals on fitted values, revealing no strong evidence of variance heterogeneity.

All statistical analyses were conducted using Stata Statistical Software: Release 18 (StataCorp, College Station, TX, USA). A two-sided significance level of 0.05 was used for all hypothesis tests.

### 2.4. Ethical Considerations

The Human Research Ethics Committee of Thammasat University (Science) granted this study an exemption from requiring ethics approval on 21 January 2024 (Document No. COE 005/2567, Research Project Code: 66PH170). Given the retrospective study design and minimal risk to participants, the ethics committee waived the requirement for informed consent. All data were fully anonymized, and data collection, access, and handling were conducted in accordance with institutional regulations and applicable ethical guidelines to ensure patient confidentiality.

## 3. Results

### 3.1. Baseline Characteristics of Participants

**Figure 1:**
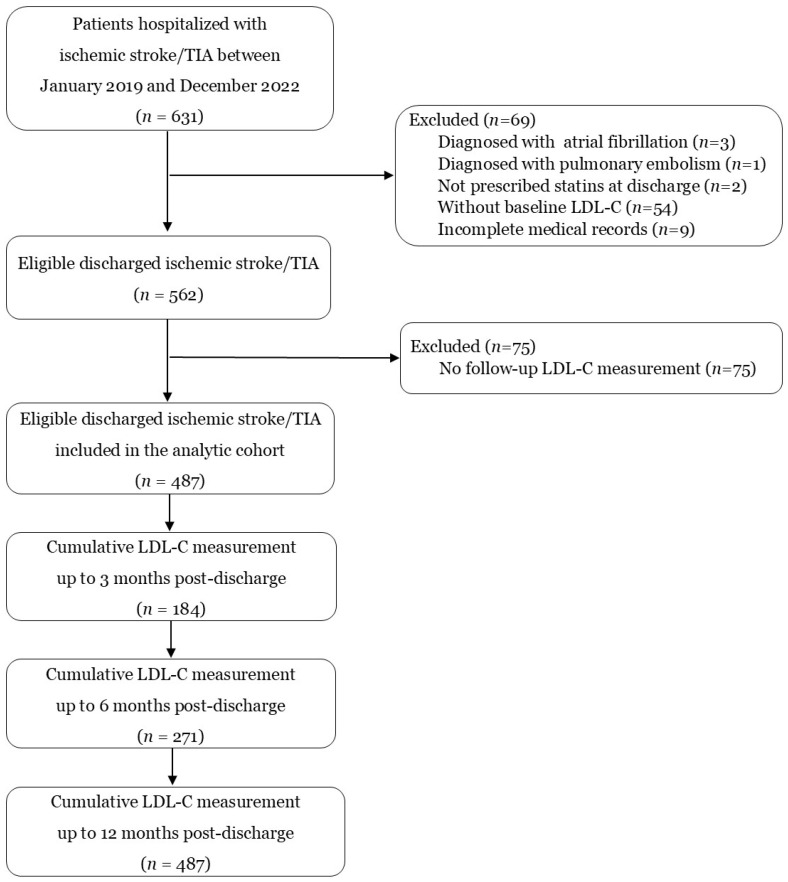
Participant flow diagram showing patient selection and LDL-C testing coverage. Of 631 ischemic stroke/TIA admissions between January 2019 and December 2022, 562 were eligible after exclusions, and 487 (86.7%) had at least one follow-up LDL-C measurement within 12 months post-discharge. LDL-C testing proportions were cumulative at 3, 6, and 12 months, such that patients tested earlier were included in later intervals.

As shown in Figure 1, a total of 631 patients were hospitalized with ischemic stroke or TIA between January 2019 and December 2022. After applying the exclusion criteria, 562 eligible patients were discharged during the study period. Within 3 months after discharge, 184 patients (32.7%) had undergone at least one LDL-C measurement, increasing to 271 patients (48.2%) by 6 months and reaching 487 patients (86.7%) within 12 months. Only 487 eligible patients who had at least one LDL-C measurement within 12 months were included in the analytic cohort for subsequent evaluation of LDL-C goal attainment and its determinants.

Table 1 summarizes the baseline characteristics of the analytic cohort. The median age was 64 years (IQR 54–73), and 60.6% of the participants were 60 years or older. Nearly half of the participants were male (49.5%). The majority (85.8%) were diagnosed with ischemic stroke, while 14.2% had a TIA. The median National Institutes of Health Stroke Scale (NIHSS) score was 3 (IQR 2–5), and 25.3% of participants were current smokers. Hypertension (75.6%) and DM (34.1%) were the most prevalent comorbidities, while CKD (7.8%) and CAD (3.7%) were less prevalent. The median baseline LDL-C level was 122 mg/dL (IQR 92–155).

Among the study participants, 74.9% (365/487) were prescribed high-intensity statin therapy, all of whom received atorvastatin 40 mg daily, which represented the highest potency statin available in the hospital formulary. No patients received atorvastatin 80 mg daily, and rosuvastatin was not used during the study period. Moderate-intensity statins were prescribed in 22.2% (108/487) of cases—most commonly simvastatin 20 mg daily (20.4%), followed by atorvastatin 20 mg daily (1.2%) and simvastatin 40 mg daily (0.6%)—while only 2.9% (14/487) of patients were discharged on low-intensity statin therapy. None of the participants were prescribed adjunct lipid-lowering therapy such as ezetimibe or proprotein convertase subtilisin/kexin type 9 (PCSK9) inhibitors, reflecting routine clinical practice in the study setting, where combination therapy is seldom initiated at hospital discharge.

Regarding follow-up lipid monitoring (Table 1), the timing of LDL-C reassessment after discharge varied. LDL-C was measured in 184 patients (37.8%) within 1–3 months, 87 (17.9%) within 4–6 months, and 216 (44.3%) within 7–12 months. The mean (SD) time to first LDL-C measurement was 6.4 (4.1) months, with a median of 6 months (IQR 3–11). These findings indicate that post-discharge lipid testing was often delayed beyond the 3-month guideline-recommended interval for treatment evaluation and dose adjustment.

### 3.2. Prevalence of LDL-C Goal Attainment

**Figure 2:**
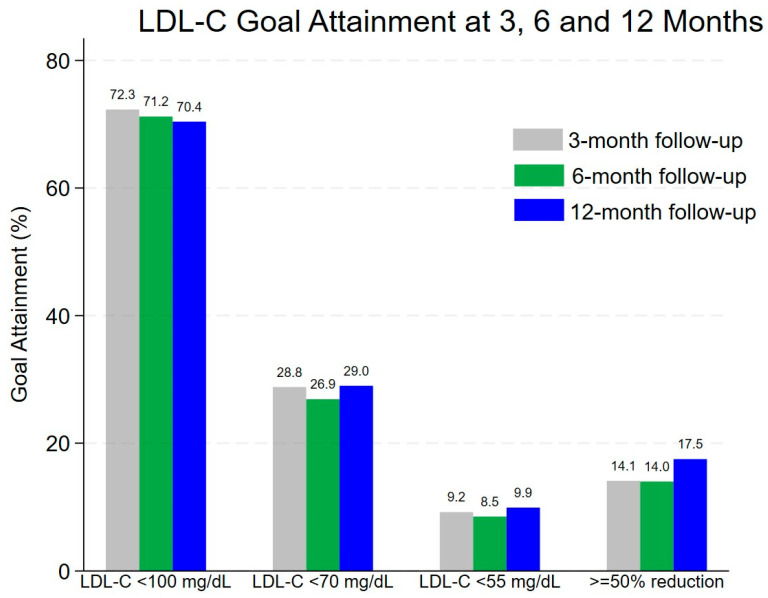
Visual representation of LDL-C goal attainment at 3, 6, and 12 months post-discharge. This figure complements the results in Table 2 by illustrating the percentage of ischemic stroke/TIA patients who achieved LDL-C targets of <100 mg/dL, <70 mg/dL, <55 mg/dL, or a ≥50% reduction from baseline. Goal attainment is shown for patients with LDL-C measured within 3 months (gray bars, *n* = 184), within 6 months (green bars, *n* = 271), and within 12 months (blue bars, *n* = 487) after hospital discharge. Denominators are cumulative across time points, and proportions are conditional on having an LDL-C test performed. LDL-C, low-density lipoprotein cholesterol.

To estimate the prevalence of LDL-C goal attainment, we determined the proportion of patients who reached LDL-C target conditional on being tested by the specified time, i.e., within 3, 6, and 12 months after discharge. Table 2 reveals that within 3 months post-discharge, 184 patients had their LDL-C measurements, and only 17 patients (9.2%, 95%CI: 5.5–14.4%) achieved an LDL-C goal of <55 mg/dL, while 28.8% (53/184) and 72.3% (133/184) reached < 70 mg/dL and <100 mg/dL, respectively. Only 26 of 184 patients (14.1%, 95%CI: 9.4–20.0%) achieved a reduction of 50% or more from baseline within 3 months. By 12 months, these proportions remained similar, with 9.9% (48/487), 29.0% (141/487), and 70.4% (343/487) achieving LDL-C < 55 mg/dL, <70 mg/dL, and <100 mg/dL, respectively. The proportion of patients who achieved a ≥50% LDL-C reduction approached 17.5% (85/487, 95%CI: 14.2–21.1%) within 12 months. Overall, the proportion of patients achieving stricter LDL-C targets (<55 mg/dL, <70 mg/dL, and 50% reduction from baseline) remained low throughout the follow-up period; however, most patients (>70%) consistently maintained LDL-C < 100 mg/dL (Figure 2).

### 3.3. Determinants of LDL-C Goal Attainment

Table 3 presents the results of the multivariable logistic regression analysis, assessing the association between potential predictors and LDL-C goal attainment within 12 months after discharge. Patients receiving high-intensity statins were significantly more likely to achieve LDL-C levels of <70 mg/dL (adjusted OR: 1.91, 95% CI: 1.09–3.34, *p* = 0.023), <100 mg/dL (adjusted OR: 1.64, 95% CI: 1.01–2.67, *p* = 0.045), and ≥50% reduction in LDL-C (adjusted OR: 2.32, 95% CI: 1.14–4.73, *p* = 0.020), compared with those receiving moderate- or low-intensity statins. However, the association between high-intensity statin use and achieving the most stringent LDL-C goal of <55 mg/dL did not reach statistical significance (adjusted OR: 1.65, 95% CI: 0.72–3.79, *p* = 0.237).

Among other covariates, baseline LDL-C level ≥ 100 mg/dL was a strong determinant of LDL-C goal attainment, with a markedly increased likelihood of achieving a ≥50% LDL-C reduction (adjusted OR: 12.13, 95% CI: 4.31–34.15, *p* < 0.001), indicating that patients with higher baseline LDL-C levels were significantly more likely to achieve a substantial percentage reduction. However, patients with a baseline LDL-C level of ≥100 mg/dL were significantly less likely to attain absolute LDL-C targets of <55 mg/dL, <70 mg/dL, and <100 mg/dL (*p* < 0.001 for all), reflecting the challenge of attaining strict targets despite substantial relative reductions. In addition, DM was significantly associated with achieving a ≥50% reduction in LDL-C from baseline (adjusted OR 1.75, 95% CI 1.03–2.98, *p* = 0.040). No statistically significant associations were observed for age, sex, smoking status, CKD, or CAD in any LDL-C goal levels.

To further quantify the determinants of LDL-C change as a continuous outcome, GLM with a Gaussian distribution and an identity link function was applied. This analysis confirmed and complemented the logistic regression findings by estimating the mean percentage reduction in LDL-C associated with each predictor while adjusting for potential confounding baseline characteristics (Table 4). The GLM demonstrated that both statin intensity and baseline LDL-C levels were independent predictors of the magnitude of LDL-C reduction over a 12-month period. High-intensity statins were significantly associated with a greater LDL-C reduction, with an estimated 6.27% greater reduction compared to non-high-intensity statins (β = 6.27, 95% CI: 1.51–11.05, *p* = 0.010). Similarly, a baseline LDL-C level greater than 100 mg/dL was identified as the strongest predictor of LDL-C reduction, showing an estimated 30.37% greater reduction (β = 30.37, 95% CI: 24.50–36.23, *p* < 0.001). These findings reinforce the key roles of both statin potency and initial LDL-C burden in determining the magnitude of lipid lowering. Other baseline characteristics, including male gender, age ≥ 60 years, DM, CKD, CAD, and smoking status, were not significantly associated with LDL-C reduction (*p* > 0.05 for all).

## 4. Discussion

Our study highlights the variability in LDL-C goal attainment among ischemic stroke/TIA patients following statin therapy initiation during the index admission. The findings from Table 2 and Figure 2 demonstrate that while a majority of patients (70.4%) reached the LDL-C target of <100 mg/dL within 12 months, attainment of more stringent goals, such as <70 mg/dL (29.0%) and <55 mg/dL (9.9%), remained suboptimal. These results highlight the challenge of achieving lower LDL-C thresholds in secondary prevention, aligning with previous reports that suggest more intensive lipid-lowering strategies may be necessary for high-risk patients. Additionally, only 17.5% of patients achieved a ≥50% reduction from baseline LDL-C, further underscoring the need for treatment optimization.

Previous studies also disclosed the suboptimal achievement of LDL-C goals in patients with ASCVD or at very high cardiovascular risk [13,14,15,16]. A study conducted in Spain reported that 25% of patients with high and very high cardiovascular risk achieved LDL-C targets based on 2019 guidelines proposed by the ESC/EAS [3,13]. In a large cohort of ASCVD patients enrolled in the commercial and Medicare programs in the United States, less than 20% of patients with baseline LDL-C > 70 mg/dL achieved LDL-C < 70 mg/dL within 12 months of follow-up [14]. Among them, high-intensity statins were prescribed to 42% of all patients. A study in a Danish cohort reported that 50% of ASCVD patients achieved the LDL-C goal of <70 mg/dL [15]. A previous study in Thailand also reported that 20% of patients with ASCVD achieve LDL-C < 70 mg/dL, with ischemic stroke patients accounting for 10% of the study population [16]. Collectively, these studies highlight the suboptimal LDL-C attainment among patients with ASCVD or very high cardiovascular risk.

A few studies specifically addressed LDL-C goal attainment after acute ischemic stroke/TIA [9,10]. A cross-sectional study in the Chinese population reported that 27% of ischemic stroke patients achieved LDL-C goals < 70 mg/dL [9]. A study of ischemic stroke patients in Israel found that 42% and 23% of the cohort achieved the LDL-C targets of <70 and 55 mg/dL, respectively [10]. The results of our study in Thai patients further confirmed these findings, with 30% and 10% of the ischemic stroke/TIA patients achieving LDL-C goals < 70 and <55 mg/dL within 12 months after discharge with acute ischemic stroke/TIA, respectively. These further underscore the need for policymakers to increase awareness among healthcare providers about the issue and to establish LDL-C targets that are suitable for this patient population.

The significance of achieving LDL-C targets to prevent recurrent ischemic stroke cannot be overstated. The Treat Stroke to Target trial demonstrated the benefits of reaching a target of LDL-C < 70 mg/dL compared to the target of 90–110 mg/dL [6]. In a separate post hoc analysis, the results underscored the importance of achieving a lower LDL-C target (<70 mg/dL) and a reduction in LDL-C by more than 50% from baseline to experience a significantly lower risk of atherosclerotic vascular events [7]. A meta-analysis of randomized controlled trials found that more intensive LDL-C-lowering statin-based therapies were significantly associated with a lower risk of recurrent stroke compared to less intensive strategies [17]. The benefits of intensive lipid-lowering therapy were pronounced in ischemic stroke patients having evidence of atherosclerosis [17]. In an observational study, good statin adherence after ischemic stroke/TIA was associated with lower recurrent ischemic stroke and acute coronary events [18]. The discontinuation of statin therapy after discharge was associated with a higher hazard of all-cause mortality after 1 year, further emphasizing the benefit of LDL-C lowering with statins after acute ischemic stroke/TIA [19].

Our study revealed that high-intensity statin therapy was significantly associated with higher odds of achieving LDL-C targets of <70 mg/dL and <100 mg/dL, as well as a ≥50% reduction from baseline (Table 3). However, it did not significantly increase the likelihood of attaining the most stringent LDL-C goal of <55 mg/dL. These findings indicate that although high-intensity statins effectively lower LDL-C levels, monotherapy may be insufficient to achieve very low LDL-C thresholds recommended for secondary prevention in high-risk patients. Similar observations have been reported in real-world cohorts, where only a small proportion of patients achieved a level of <55 mg/dL despite receiving high-intensity statin therapy [13,20,21]. This limitation is particularly evident among individuals with high baseline LDL-C concentrations, who require greater absolute reductions to reach target levels. Therefore, the addition of non-statin lipid-lowering agents, such as ezetimibe or PCSK9 inhibitors, should be considered to optimize treatment outcomes. Such combination therapy has demonstrated a substantial incremental reduction in LDL-C and improved cardiovascular protection in patients with atherosclerotic disease, including ischemic stroke and TIA [6,7].

The high-intensity statin prescribed to the ischemic stroke/TIA patients in our study was atorvastatin 40 mg daily. The atorvastatin employed in the SPARCL trial was 80 mg daily [4]. The use of a lower dose of high-intensity atorvastatin reflected the evidence that the Asian population has higher statin plasma concentrations than Westerners, possibly a lower dose requirement for therapeutic effect, and a lower risk of adverse effects [22,23,24,25]. The possible explanation lies in genetic variation in drug-metabolizing enzymes and transporters (e.g., *SLCO1B1*, *ABCG2*), which affect statin metabolism and clearance [23]. Thus, the suboptimal LDL-C goal attainment observed in our study is unlikely to be attributed solely to the lower high-intensity atorvastatin dose, but rather to other factors. Nevertheless, the possibility that this dosing practice may have contributed to the limited proportion of patients achieving the target of <55 mg/dL, despite being on high-intensity statins, cannot be excluded. Our findings of suboptimal LDL-C goal achievement remain consistent with prior studies in ASCVD and ischemic stroke patients [9,10,13,14,15,16].

In the present study, DM was independently associated with achieving an LDL-C reduction of 50% or more from baseline. This observation is consistent with previous studies reporting that patients with type 2 DM tend to have lower LDL-C concentrations and a higher likelihood of achieving lipid-lowering targets compared with non-diabetic individuals [26,27]. Several mechanisms may underlie this pattern. Diabetes is characterized by complex alterations in lipid metabolism, including increased LDL receptor activity and enhanced clearance of circulating LDL particles in some individuals receiving intensive glycemic control or combination therapy with lipid-lowering agents. In addition, diabetic patients may benefit from more intensive medical surveillance and improved treatment adherence through closer follow-up and multifactorial cardiovascular risk management, which could contribute to achieving the LDL-C goal. Nonetheless, the association observed in our study was modest and not consistent across all LDL-C goal levels. Therefore, unmeasured factors such as adherence, diet, or disease severity may have influenced the results, and this finding should be interpreted with caution.

Demographic and clinical factors, including DM, CKD, CAD, older age, and male gender, showed a trend toward reduced LDL-C lowering after adjusting for statin intensity and baseline LDL-C (Table 3). Although these associations were not statistically significant, future research should investigate the underlying mechanisms contributing to these variations in patients with ischemic stroke/TIA. Factors not accounted for in this study, such as genetic polymorphisms, medication adherence, and metabolic differences, may influence statin response. Incorporating pharmacogenomic and adherence data in future studies could help optimize lipid-lowering strategies for this patient population.

The study period (January 2019 to December 2022) overlapped with the COVID-19 pandemic, during which several studies reported transient reductions in serum lipid levels, particularly in LDL-C and total cholesterol, among patients infected with SARS-CoV-2 [28,29]. These alterations have been attributed to systemic inflammation, cytokine-mediated changes in hepatic lipid metabolism, and increased catabolism of apolipoproteins during acute infection. Such lipid disturbances generally normalize after recovery and are more pronounced in patients with severe disease. In our cohort, none of the included patients were hospitalized with confirmed SARS-CoV-2 infection during the study period. Furthermore, LDL-C levels were measured during scheduled outpatient follow-up visits rather than during acute illness. Therefore, it is unlikely that the transient, infection-related lipid changes associated with COVID-19 significantly influenced our results. Nevertheless, given the global impact of the pandemic, subtle indirect effects, such as reduced healthcare access or delays in follow-up testing, cannot be entirely excluded and should be considered when interpreting our findings.

In addition to pharmacological management, lipid control in post-stroke and TIA patients is strongly influenced by lifestyle modifications, including dietary patterns, physical activity, weight control, and smoking cessation [30]. Evidence suggests that adhering to a heart-healthy diet, which includes reducing saturated fat intake and increasing consumption of fruits, vegetables, and whole grains, can contribute to additional LDL-C lowering beyond that achieved through pharmacotherapy. Regular physical activity and smoking cessation have also been shown to improve lipid metabolism and overall cardiovascular outcomes. Unfortunately, detailed information on these behavioral factors and the use of complementary therapies was not routinely captured in the medical records used for this study. Consequently, their potential impact on achieving the LDL-C goal could not be assessed. Future prospective studies that incorporate both pharmacologic and non-pharmacologic management data are warranted to provide a more comprehensive understanding of lipid control strategies and to guide individualized secondary prevention programs after stroke and TIA.

This study has limitations. Its observational design prevents causal inference of the association between determinants and the LDL-C goal attainment. Among ischemic stroke/TIA patients discharged during the study period, 75 were excluded from the analysis due to the absence of follow-up LDL-C testing, leaving 487 of 562 patients (86.7%) with at least one LDL-C measurement within 12 months after discharge. This exclusion may have introduced selection bias, as patients who did not undergo LDL-C testing could differ in characteristics influencing LDL-C goal attainment in either direction. Therefore, our findings should be interpreted with caution in light of this potential limitation. Additionally, adherence to statin therapy, diet, and over-the-counter products was not fully accounted for, potentially influencing LDL-C goal attainment. Future studies should explore patient adherence and strategies to optimize LDL-C control in post-discharge stroke/TIA patients. Lastly, as our findings are based on a single-center study, their generalizability to other settings and populations requires validation. However, our results align with and support previous research [9,10].

In conclusion, LDL-C goal attainment remains suboptimal after acute ischemic stroke/TIA, particularly for the lower LDL-C targets. High-intensity statins significantly improve the likelihood of achieving moderate LDL-C goals and LDL-C reductions but may be insufficient for the most stringent targets. These findings underscore the importance of enhancing healthcare provider awareness and considering combination lipid-lowering therapies to improve secondary prevention efforts.

## Figures and Tables

**Table 1:** Baseline characteristics of the study participants (N = 487).

Characteristics	Values
Age (years)—median (IQR)	64 (54–73)
Age ≥ 60 years—no. (%)	295 (60.6)
Male—no. (%)	241 (49.5)
Body mass index (kg/m^2^)—median (IQR)	24.1 (21.3–27.1)
Principal Diagnosis—no. (%)	
• Ischemic stroke	418 (85.8)
• Transient ischemic attack	69 (14.2)
NIHSS score—median (IQR) (*n* = 388)	3 (2–5)
ABCD^2^ score—median (IQR) (*n* = 63)	4 (3–5)
Current smoking—no. (%)	123 (25.3)
Comorbidities—no. (%)	
• Hypertension	368 (75.6)
• Diabetes mellitus	166 (34.1)
• Chronic kidney disease	38 (7.8)
• Coronary artery disease	18 (3.7)
Glycated hemoglobin (mg/dL)—median (IQR)	7.2 (5.9–7.2)
Serum creatinine (mg/dL)—median (IQR)	0.9 (0.7–1.1)
Baseline lipid profile (mg/dL)—median (IQR)	
• Total cholesterol	197 (163–235)
• LDL-C	122 (92–155)
• HDL-C	45 (36–53)
• Triglyceride	113 (78–170)
Discharged statins—no. (%)	
• Low–intensity statins (simvastatin 10 mg daily)	14 (2.9)
• Moderate–intensity statins	108 (22.2)
• Atorvastatin 20 mg daily	6 (1.2)
• Simvastatin 40 mg daily	3 (0.6)
• Simvastatin 20 mg daily	99 (20.4)
• High–intensity statins (atorvastatin 40 mg daily)	365 (74.9)
Other discharge medications—no. (%)	
• Aspirin	477 (97.9)
• Clopidogrel	188 (38.6)
• Beta-blockers	26 (5.3)
• ACEIs or ARBs	113 (23.2)
Time of LDL-C measurement post-discharge—no. (%)	
• 1 to 3 months	184 (37.8)
• 4 to 6 months	87 (17.9)
• 7 to 12 months	216 (44.3)
Time to first LDL-C measurement—mean (SD)	6.4 (4.1)
Time to first LDL-C measurement—median (IQR)	6 (3–11)

Abbreviations: ABCD^2^, age, blood pressure, clinical features of transient ischemic attack, duration of symptoms, and diabetes; ACEIs, angiotensin-converting-enzyme inhibitors; ARBs, angiotensin receptor blockers; NIHSS, National Institute of Health Stroke Scale.

**Table 2:** Prevalence of LDL-C goal attainment after discharge.

LDL-C Goal	LDL-C Goal Attainment After Discharge * *n* (%, 95%CI) **		
	Within 3 Months (*N* = 184)	Within 6 Months (*N* = 271)	Within 12 Months (*N* = 487)
LDL-C < 100 mg/dL	133 (72.3, 65.2–78.6)	193 (71.2, 65.4–76.5)	343 (70.4, 66.2–74.5)
LDL-C < 70 mg/dL	53 (28.8, 22.4–35.9)	73 (26.9, 21.7–32.6)	141 (29.0, 25.0–33.2)
LDL-C < 55 mg/dL	17 (9.2, 5.5–14.4)	23 (8.5, 5.5–12.5)	48 (9.9, 7.4–12.9)
≥50% LDL-C reduction from baseline	26 (14.1, 9.4–20.0)	38 (14.0, 10.1–18.7)	85 (17.5, 14.2–21.1)

* Denominators represent the cumulative number of patients who underwent LDL-C testing up to each specified period. By 12 months, the denominator encompasses the entire cohort (*N* = 487). ** Numbers in parentheses represent percentages of LDL-C goal attainment and their 95%CIs.

**Table 3:** Determinants of LDL-C goal attainment within 12 months in multivariable logistic regression analysis.

Variables *	LDL-C Goal Attainment Within 12 Months							
	<100 mg/dL		<70 mg/dL		<55 mg/dL		≥50% Reduction from Baseline	
	Adjusted OR (95%CI)	*p*-Value	Adjusted OR (95%CI)	*p*-Value	Adjusted OR (95%CI)	*p*-Value	Adjusted OR (95%CI)	*p*-Value
High-intensity statin	1.64 (1.01–2.67)	0.045	1.91 (1.09–3.34)	0.023	1.65 (0.72–3.79)	0.237	2.32 (1.14–4.73)	0.020
Age ≥ 60 years	1.25 (0.82–1.90)	0.305	1.22 (0.78–1.92)	0.388	1.17 (0.59–2.32)	0.656	1.11 (0.66–1.88)	0.683
Male	1.24 (0.79–1.96)	0.345	0.70 (0.44–1.13)	0.144	0.81 (0.41–1.61)	0.542	0.90 (0.52–1.57)	0.719
Current smokers	0.60 (0.36–1.01)	0.057	1.41 (0.82–2.45)	0.219	1.04 (0.45–2.43)	0.922	1.10 (0.58–2.07)	0.775
DM	0.75 (0.48–1.16)	0.192	0.89 (0.56–1.42)	0.630	1.26 (0.65–2.45)	0.489	1.75 (1.03–2.98)	0.040
CKD	1.48 (0.63–3.45)	0.364	0.94 (0.38–2.35)	0.898	2.04 (0.65–6.41)	0.225	0.50 (0.13–1.89)	0.310
CAD	0.41 (0.14–1.17)	0.094	0.52 (0.15–1.76)	0.293	0.69 (0.14–3.41)	0.646	0.69 (0.14–3.46)	0.650
LDL-C ≥ 100 mg/dL	0.31 (0.19–0.52)	<0.001	0.17 (0.11–0.27)	<0.001	0.13 (0.07–0.26)	<0.001	12.13 (4.31–34.15)	<0.001

* The baseline categories of all predictors were coded as 0, and the characteristics indicated in the Table were coded as 1 in the statistical analysis. Abbreviations: CAD, history of coronary artery disease; CKD, chronic kidney disease; DM, diabetes mellitus.

**Table 4:** Determinants of the percentage of LDL-C reduction from baseline within 12 months in the generalized linear model (*N* = 487).

Variables *	Coefficients ** (% LDL-C Reduction)	95%CIs	z	*p*-Value
High-intensity statin	6.27	(1.51, 11.05)	2.14	0.010
Age ≥ 60 years	−3.45	(−7.91, 1.00)	−1.52	0.128
Male	−2.59	(−7.36, 2.19)	−1.06	0.288
Current smokers	−3.07	(−9.01, 2.87)	−1.01	0.311
DM	−5.32	(−10.80, 0.15)	−1.90	0.057
CKD	−1.07	(−12.62, 10.48)	−0.18	0.856
CAD	−8.73	(−22.98, 5.51)	−1.20	0.230
Baseline LDL-C > 100 mg/dL	30.37	(24.50, 36.23)	10.15	<0.001
Intercept	5.12	(−1.48, 11.72)	1.52	0.129

Model: Gaussian family with identity link function, log pseudolikelihood = −2277.97, and robust standard errors were used to account for heteroscedasticity. * The baseline categories of all predictors were coded as 0, and the characteristics indicated in the Table were coded as 1 in the statistical analysis. ** The negative coefficients indicate that the independent variable induced an increase in LDL-C (%), whereas the positive coefficients indicate a reduction in LDL-C. Abbreviations: CAD, coronary artery disease; CKD, chronic kidney disease; DM, diabetes mellitus.

## Data Availability

The data presented in this study are available on request from the corresponding author due to institutional and ethical restrictions. The dataset was obtained from hospital electronic medical records under a data-use agreement that permits analysis for this specific research only; the hospital does not authorize public dissemination of patient-level data. De-identified data may be provided upon reasonable request and with institutional approval.

## References

[1] Katan M., Luft A. (2018). Global burden of stroke. Semin. Neurol..

[2] Kleindorfer D.O., Towfighi A., Chaturvedi S., Cockroft K.M., Gutierrez J., Lombardi-Hill D., Kamel H., Kernan W.N., Kittner S.J., Leira E.C. (2021). 2021 Guideline for the prevention of stroke in patients with stroke and transient ischemic attack: A guideline from the American Heart Association/American Stroke Association. Stroke.

[3] Authors/Task Force Members, ESC Committee for Practice Guidelines (CPG), ESC National Cardiac Societies (2019). 2019 ESC/EAS guidelines for the management of dyslipidaemias: Lipid modification to reduce cardiovascular risk. Atherosclerosis.

[4] Amarenco P., Bogousslavsky J., Callahan A., Goldstein L.B., Hennerici M., Rudolph A.E., Sillesen H., Simunovic L., Szarek M., Welch K.M.A. (2006). High-dose atorvastatin after stroke or transient ischemic attack. N. Engl. J. Med..

[5] Pan Y., Wangqin R., Li H., Jin A., Li J., Lin J., Meng X., Xian Y., Laskowitz D.T., Wang Y. (2022). LDL-C levels, lipid-lowering treatment and recurrent stroke in minor ischaemic stroke or TIA. Stroke Vasc. Neurol..

[6] Amarenco P., Kim J.S., Labreuche J., Charles H., Abtan J., Béjot Y., Cabrejo L., Cha J.-K., Ducrocq G., Giroud M. (2020). A comparison of two LDL cholesterol targets after ischemic stroke. N. Engl. J. Med..

[7] Amarenco P., Lavallée P.C., Kim J.S., Labreuche J., Charles H., Giroud M., Lee B.-C., Mahagne M.-H., Meseguer E., Nighoghossian N. (2023). More than 50 percent reduction in LDL cholesterol in patients with target LDL < 70 mg/dL after a stroke. Stroke.

[8] Chen K.N., He L., Zhong L.M., Ran Y.Q., Liu Y. (2020). Meta-analysis of dyslipidemia management for the prevention of ischemic stroke recurrence in China. Front. Neurol..

[9] Wang C.J., Wang Y.L., Li Z.X., Wang Y.J. (2016). The management of LDL cholesterol and predictors of goal achievement in stroke patients in China: A cross-sectional study. CNS Neurosci. Ther..

[10] Zafrir B., Aker A., Naoum I., Saliba W. (2023). Guideline-directed low-density lipoprotein cholesterol management after acute ischemic stroke: Findings from a national health care service. Am. J. Cardiol..

[11] Barrios V., Pintó X., Escobar C., Varona J.F., Gámez J.M. (2023). Real-world attainment of low-density lipoprotein cholesterol goals in patients at high risk of cardiovascular disease treated with high-intensity statins: The TERESA Study. J. Clin. Med..

[12] Wongsalap Y., Jedsadayanmata A. (2020). Trends and predictors of high-intensity statin therapy and LDL-C goal achievement among Thai patients with acute coronary syndrome. J. Cardiol..

[13] Mostaza J.M., García-Ortiz L., Suárez Tembra M.A., Calle P.T., García J.C., Pérez V.E., Díaz-Díaz J., Manzano-Espinosa L., Catapano A., Ray K. (2025). Failure of LDL-C goals achievement and underuse of lipid-lowering therapies in patients at high and very high cardiovascular risk: Spanish subset from the European SANTORINI study. Rev. Clin. Esp..

[14] Navar A.M., Electricwala B., Multani J.K., Zhou Z., Chen C.-C., Agatep B.C., Petrilla A.A., Schwartz T.T., N’DRi L., Cristino J. (2025). Lipid management in United States commercial and Medicare enrollees with atherosclerotic cardiovascular disease: Treatment patterns and low-density lipoprotein cholesterol control. Am. J. Cardiol..

[15] Ersbøll A.K., Kristensen M.S., Nybo M., Hede S.M., Mikkelsen K.H., Gislason G., Larsen M.L., Green A. (2023). Trends in low-density lipoprotein cholesterol goal achievement and changes in lipid-lowering therapy after incident atherosclerotic cardiovascular disease: Danish cohort study. PLoS ONE.

[16] Krittayaphong R., Phrommintikul A., Boonyaratvej S., Na Ayudhya R.K., Tatsanavivat P., Komoltri C., Sritara P., CORE Investigators (2019). The rate of patients at high risk for cardiovascular disease with an optimal low-density cholesterol level: A multicenter study from Thailand. J. Geriatr. Cardiol..

[17] Lee M., Cheng C.Y., Wu Y.L., Lee J.D., Hsu C.Y., Ovbiagele B. (2022). Association between intensity of low-density lipoprotein cholesterol reduction with statin-based therapies and secondary stroke prevention: A meta-analysis of randomized clinical trials. JAMA Neurol..

[18] Chen P.S., Cheng C.L., Kao Yang Y.H., Li Y.H. (2016). Statin adherence after ischemic stroke or transient ischemic attack is associated with clinical outcome. Circ. J..

[19] Colivicchi F., Bassi A., Santini M., Caltagirone C. (2007). Discontinuation of statin therapy and clinical outcome after ischemic stroke. Stroke.

[20] Ray K.K., Molemans B., Schoonen W.M., Giovas P., Bray S., Kiru G., Murphy J., Banach M., De Servi S., Gaita D. (2021). EU-Wide cross-sectional observational study of lipid-modifying therapy use in secondary and primary care: The DA VINCI study. Eur. J. Prev. Cardiol..

[21] Ray K.K., Aguiar C., Arca M., Connolly D.L., Eriksson M., Ferrières J., Laufs U., Mostaza J.M., Nanchen D., Bardet A. (2024). Use of combination therapy is associated with improved LDL cholesterol management: 1-year follow-up results from the European observational SANTORINI study. Eur. J. Prev. Cardiol..

[22] Adams S.P., Tsang M., Wright J.M. (2015). Lipid-lowering efficacy of atorvastatin. Cochrane Database Syst. Rev..

[23] Liao J.K. (2007). Safety and efficacy of statins in Asians. Am. J. Cardiol..

[24] Tomlinson B., Chan P., Liu Z.M. (2020). Statin intolerance-an Asian perspective. J. Atheroscler. Thromb..

[25] Li Y.F., Feng Q.Z., Gao W.Q., Zhang X.J., Huang Y., Chen Y.D. (2015). The difference between Asian and Western in the effect of LDL-C lowering therapy on coronary atherosclerotic plaque: A meta-analysis report. BMC Cardiovasc. Disord..

[26] Saely C.H., Eber B., Pfeiffer K.P., Drexel H., LIIFE-IN-LIFE study group (2010). Low serum LDL cholesterol in patients with type 2 diabetes: An analysis on two different patient populations. Int. J. Cardiol..

[27] Saely C.H., Sternbauer S., Vonbank A., Heinzle C., Zanolin-Purin D., Larcher B., Mader A., Leiherer A., Muendlein A., Drexel H. (2020). Type 2 diabetes mellitus is a strong predictor of LDL cholesterol target achievement in patients with peripheral artery disease. J. Diabetes Complicat..

[28] Aparisi Á., Martín-Fernández M., Ybarra-Falcón C., Gil J.F., Carrasco-Moraleja M., Martínez-Paz P., Cusácovich I., Gonzalo-Benito H., Fuertes R., Marcos-Mangas M. (2022). Dyslipidemia and inflammation as hallmarks of oxidative stress in COVID-19: A follow-up study. Int. J. Mol. Sci..

[29] Kowalska K., Sabatowska Z., Forycka J., Młynarska E., Franczyk B., Rysz J. (2022). The influence of SARS-CoV-2 infection on lipid metabolism-the potential use of lipid-lowering agents in COVID-19 management. Biomedicines.

[30] Govori V., Budinčević H., Morović S., Đerke F., Demarin V. (2024). Updated perspectives on lifestyle interventions as secondary stroke prevention measures: A narrative review. Medicina.

